# Contextual Patch-NetVLAD: Context-Aware Patch Feature Descriptor and Patch Matching Mechanism for Visual Place Recognition

**DOI:** 10.3390/s24030855

**Published:** 2024-01-28

**Authors:** Wenyuan Sun, Wentang Chen, Runxiang Huang, Jing Tian

**Affiliations:** 1 Institute of Systems Science, National University of Singapore, Singapore 119615, Singapore; e0983371@u.nus.edu (W.S.); e0983021@u.nus.edu (R.H.); 2 State Key Laboratory of Fluid Power and Mechatronic Systems, School of Mechanical Engineering, Zhejiang University, Hangzhou 310027, China; 22125186@zju.edu.cn; 3 Engineering Research Center for Design Engineering and Digital Twin of Zhejiang Province, School of Mechanical Engineering, Zhejiang University, Hangzhou 310027, China; 4 Robotics Institute, Zhejiang University, Hangzhou 310027, China

**Keywords:** visual place recognition, feature description, feature learning, feature matching

## Abstract

The goal of *visual place recognition* (VPR) is to determine the location of a query image by identifying its place in a collection of image databases. Visual sensor technologies are crucial for visual place recognition as they allow for precise identification and location of query images within a database. Global descriptor-based VPR methods face the challenge of accurately capturing the local specific regions within a scene; consequently, it leads to an increasing probability of confusion during localization in such scenarios. To tackle feature extraction and feature matching challenges in VPR, we propose a modified patch-NetVLAD strategy that includes two new modules: a *context-aware patch descriptor* and a *context-aware patch matching* mechanism. Firstly, we propose a context-driven patch feature descriptor to overcome the limitations of global and local descriptors in visual place recognition. This descriptor aggregates features from each patch’s surrounding neighborhood. Secondly, we introduce a context-driven feature matching mechanism that utilizes cluster and saliency context-driven weighting rules to assign higher weights to patches that are less similar to densely populated or locally similar regions for improved localization performance. We further incorporate both of these modules into the patch-NetVLAD framework, resulting in a new approach called *contextual patch-NetVLAD*. Experimental results are provided to show that our proposed approach outperforms other state-of-the-art methods to achieve a Recall@10 score of 99.82 on Pittsburgh30k, 99.82 on FMDataset, and 97.68 on our benchmark dataset.

## 1. Introduction

The sensing technique plays a pivotal role in place recognition [1,2], where the goal is to estimate the location of input query data from a reference database. An important aspect of such a perception-based application is the selection of appropriate sensors based on sensor capabilities and application specificities. The commonly used sensors for this application are cameras, LiDAR, and RADAR [3]. Camera-based image sensing technology is critical in place recognition due to its ability to capture detailed visual data [4,5]. Compared to other sensing methods like LiDAR and RADAR, image sensors can provide rich color information, which is crucial for distinguishing between similar structures or landscapes. They provide high precision in identifying and locating query images within a database. Moreover, they are also generally less expensive and easier to implement. For instance, in autonomous vehicles, image sensors contribute to the recognition of traffic signs, pedestrians, and other vehicles.

The two major challenges of *visual place recognition* (VPR) are feature description and feature matching. Firstly, in traditional VPR techniques, local features like SIFT [6], SURF [7], and ORB [8] are manually crafted and can be combined into a global descriptor, such as VLAD [9], or a convolutional neural network (CNN)-based NetVLAD [10]. Nevertheless, local features face challenges in handling significant changes in illumination. An alternative approach is to apply spatial pooling from the feature map of the backbone model to examine regions of interest. For example, a sliding window technique is utilized to generate patches, and patch descriptors are subsequently derived from NetVLAD [11]. Secondly, one common feature-matching approach is to exploit a two-stage retrieval strategy. This strategy involves performing a global retrieval step to retrieve a set of top candidates from the reference database for each query. These candidates are then refined in a subsequent step based on their local features for improved image ranking.

Patch-NetVLAD [11] exploits a two-stage retrieval strategy. It applies the NetVLAD [10] to obtain descriptors to retrieve the top 100 images that are most similar to the query image and then rearrange them through patch-level matching. Patch-NetVLAD+ [12] applies a fine-tuned NetVLAD to extract patch-level descriptors and assigns weights to patches according to the distances of patch descriptors from the centroids in the description space. However, they suffer from two problems. Firstly, local descriptors emphasize spatial accuracy but may not fully represent the larger context. On the other hand, global descriptors are robust to appearance and lighting noise but struggle to identify minor local variations. Secondly, descriptors extracted from locally similar regions in descriptor space often exhibit strong similarities and are challenging to distinguish.

To tackle these inherent challenges of VPR, we propose a new VPR approach in this paper called “contextual patch-NetVLAD”. As indicated by its name, building upon the same strategy as patch-NetVLAD [11], we introduce two new modules, including a context-aware patch descriptor and a context-aware patch matching mechanism. To perform the VPR task, the proposed approach employs a sliding window method to extract patch descriptors from NetVLAD. It leverages the use of local regions for matching by decomposing the image into patches.

The following is a summary of our contextual patch-NetVLAD’s motivations and contributions.

- Local descriptors emphasize spatial accuracy but may not adequately represent the larger environment, whereas global descriptors are resilient to appearance and light changes but have difficulty identifying minute local variations. Motivated by this, a context-driven patch feature descriptor is proposed to aggregate the features from each patch’s neighborhood.
- Descriptors taken from an area of a descriptor space that is heavily populated (e.g., generic building’s walls are all very similar) or a locally similar region (e.g., descriptors extracted from the smooth ceiling area) are deemed to be less distinctive. Inspired by this, to improve VPR performance by exploiting the distinctiveness of feature descriptors, a new context-driven feature matching mechanism is proposed. It consists of (i) a cluster context-driven weighting rule, which exploits the global information of all patch features extracted from the whole database to assign larger weights for patches far away from cluster centroids in the dataset, and (ii) a saliency context-driven weighting rule, which exploits local information of each patch by comparing it and its neighboring patches’ features and assigning larger weights for patches with a higher difference.

Furthermore, we propose integrating these two contributions in the patch-NetVLAD framework [11] for the VPR task in challenging indoor environments. We conduct experiments using our benchmark dataset to evaluate the effectiveness of this integration.

**Figure 1:**
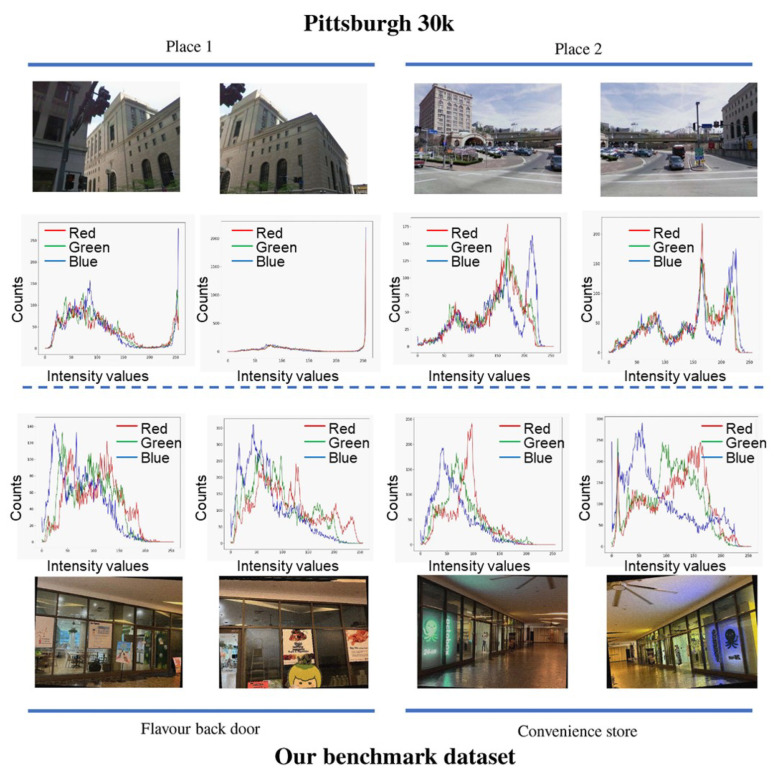
A comparison between the public Pittsburgh30k [13] dataset (the first row) and our benchmark dataset (the fourth row). The second and third lines show the color statistics (in terms of red, green, and blue histogram) of the corresponding images. The *x*-axis and *y*-axis indicate the intensity values and count of pixels, respectively.

Unlike the standard VPR datasets (e.g., Pittsburgh30k [13]) that use outdoor images, we conduct an evaluation using our benchmark indoor dataset in this paper. As shown in Figure 1, our benchmark dataset is more challenging due to three main challenges.

- *Scale*: the standard Pittsburgh30k dataset primarily comprises outdoor locations, which are typically large and spacious. In contrast, our benchmark dataset consists of indoor locations that are smaller; therefore, distinctive key features are more susceptible to obstruction from obstacles (e.g., crowded crowds).
- *Spectral Information*: as shown in Figure 1, the spectral differences between images of different locations in the standard dataset are quite significant. In contrast, the spectral information of images from different indoor locations in our benchmark dataset is relatively similar, making descriptors harder to distinguish.
- *Lighting condition*: indoor lighting in our dataset can be affected by reflections from objects like furniture and walls, leading to more intricate lighting and shadow effects than outdoor scenarios.

The rest of this paper is organized as follows. Section 2 provides a brief review of the existing VPR research works. Then, the proposed contextual path-NetVLAD is presented in Section 3, including the proposed new feature description and patch matching, and then evaluated in extensive experiments in Section 4. Finally, this paper is concluded in Section 5.

## 2. Related Works

This section provides a brief overview of VPR works with a focus on two challenges of VPR, including feature description and feature matching.

### 2.1. Feature Description

The existing feature description approaches can be grouped into three classes: (i) *global* descriptor, (ii) *local keypoint* descriptor, and (iii) *local patch* descriptor.

Firstly, *global* descriptor methods primarily focus on global statistical features of the image, such as VLAD [9], BoW [14], WI-SURF [15], and *Fish vector* (FV) [16]. In recent VPR research, deep learning methods have made significant progress by utilizing features extracted from a backbone CNN [10] that is pretrained on image classification datasets. These features are then passed through a trainable aggregation layer, which transforms them into robust and compact representations. Examples of such methods include NetBoW [17] and NetFV [18], which improve BoW [14] and FV [16] using deep learning-based architectures, respectively. Attention-based *pyramid aggregation network* (APANet) [19] utilizes spatial pyramid pooling to aggregate the multiscale information and attention blocks to highlight the discriminative features. In [20], a hybrid image descriptor is proposed to aggregate salient visual information and complement it with appearance-based descriptions. To model the saliency of local features from different dimensions, the approach incorporates three attention modules that consider individual, spatial, and cluster dimensions [21]. In the study [22], robust feature selection and matching processes are investigated to enhance the accuracy of place recognition. They integrate a BoW vocabulary with a feature matcher to adapt to varying environmental conditions. Sergi et al. explore the application of a CNN architecture to simultaneously detect and describe local features for image matching within the context of cultural heritage [23]. A novel approach termed MixVPR is introduced in [24]. It utilizes feature maps from pretrained backbones as a collection of global features and enriches them by incorporating a global relationship among elements within each feature map through a series of feature mixing stages.

Secondly, *local keypoint* descriptors primarily involve reordering the initial candidate list generated by global methods to obtain a more reasonable matching sequence [25,26,27,28]. These methods use traditional handcrafted local feature methods [6,7,8] or deep-learned local feature methods that learn discriminative and robust local features directly from images, such as LIFT [29], DeLF [30], and SuperPoint [31]. SAND features are proposed to provide hierarchical context information while extracting features [32]. However, most learning-based methods focus on enhancing nearest neighbor matching performance at the keypoint level.

Thirdly, *local patch* descriptors emphasize the local specific patch features within images instead of considering the whole image. A landmark-based VPR approach [33] is proposed to combine edge boxes [34] to detect landmarks with CNN features. A pre-trained CNN is used to re-rank the list of candidates [35] or utilize RefineNet [36] to obtain local semantic features [37]. Patch-NetVLAD [11] generates patch descriptors from the NetVLAD framework. In order to differentiate between dissimilar regions within the same scene, patch-NetVLAD+ [12] fine-tunes NetVLAD with a triplet loss to improve patch descriptor extraction for differentiating dissimilar regions within the same scene. In the study [38], a hot-spot detector is developed specifically for a learned local key-patch descriptor.

### 2.2. Feature Matching

Feature matching methods re-rank matched patch candidates, which are obtained from a global feature retrieval step; therefore, they are crucial in obtaining the final retrieval results. In the patch-NetVLAD approach [11], candidate images are initially identified using NetVLAD. Next, to rank the candidate images and identify the best matching image, the patch match score is calculated for each pair of images. However, patch-NetVLAD treats all patches equally during the matching process. To address this, a patch weighting rule is proposed in [39] that applies the standard Hamming embedding method to evaluate descriptor distinctiveness. Patch-NetVLAD+ [12] evaluates the importance of patch features and selectively assigns less frequently occurring patches a more significant role in the matching process.

## 3. Proposed Contextual Patch-Netvlad Framework

The proposed contextual patch-NetVLAD approach leverages the conventional patch-NetVLAD framework [11] with two new components proposed in this paper. Our approach involves using a sliding window technique to derive patch descriptors from NetVLAD [10]. For the query image, it is passed through backbone feature extraction layers. They are clustered into fixed clusters, and their respective residuals are obtained. This is achieved by utilizing a VLAD aggregation layer, followed by a projection layer and *principal component analysis* (PCA) to create a feature map [10].

In our proposed approach, the patch features undergo two key steps. Firstly, they are processed by the context-driven patch feature descriptor, which replaces a patch within a feature map with the average of its eight neighboring patches. This step aims to capture the contextual information within the patches. Secondly, these patch features are further processed by the context-driven patch-matching mechanism, which assigns a score to each position based on its relevance to VPR. These scores are then used as weights to adjust the contributions of the patch-matching process, taking into account the information extracted from images in the database. In the end, match scoring is carried out to calculate the similarity score between the query image and each image in the database.

### 3.1. Proposed Context-Driven Patch Feature Descriptor

Given an input query image, we build its feature map F confirm if all variables in bold should be retained. $\in R^{H\times W\times D}$ from the NetVLAD framework [10], where *H* and *W* denote the height and width of the image, respectively, and *D* is the length of the descriptor. For a patch $P_{r,c}$ centered at the *r*-th row and *c*-th column, its descriptor is represented as $f_{r,c}$. Then, rather than using its original feature $f_{r,c}$, we propose to aggregate the features from its neighborhood (as a context) to obtain its context-driven patch feature descriptor as

(1) $$f_{r,c}=\frac{1}{|\Omega_{r,c}|}\sum_{\left(m,n\right)\in \Omega_{r,c}}{\hat{f}}_{m,n},$$

where $f_{r,c}$ is the replacement of the original feature ${\hat{f}}_{r,c}$, $|\Omega_{r,c}|$ is the cardinality of the set of neighboring patches $\Omega_{r,c}$ centered at (r,c). An 8-connected neighborhood is used in our approach; therefore, (1) boils down to $f_{r,c}=\frac{1}{8}\sum_{m=r-1}^{r+1}\sum_{n=c-1}^{c+1}{\hat{f}}_{m,n}$.

By averaging a patch with its neighboring patches, we improve the representation of features within the feature map. This process effectively reduces noise and brings out more prominent features. In situations where the patch’s region extends beyond the boundaries of the feature map, automatic zero-padding is applied to fill the areas that exceed the feature map’s limits. This ensures consistent performance of the replacement operation and eliminates any boundary-related problems, allowing for uniform feature enhancement across the entire feature map.

### 3.2. Proposed Context-Driven Patch Matching

We introduce a context-driven patch-matching mechanism that incorporates two rules: (i) a cluster context-driven weighting rule, which utilizes global information from all patch features extracted from the entire database, and (ii) a saliency context-driven weighting rule, which utilizes local information of each patch by evaluating its features and those of its neighboring patches. The details of both rules are described below.

- Firstly, a cluster context-driven weighting rule is proposed. We adopt the original NetVLAD [10] to extract patch descriptor sets ${\left\{f_{i}^{db}\in R^{1\times D}\right\}}_{i=1}^{N_{p}^{db}}$ and ${\left\{f_{i}^{q}\in R^{1\times D}\right\}}_{i=1}^{N_{p}^{q}}$ from the database db and the query image *q*, respectively; *N* denotes the number of patches, and f denotes the descriptor. We take into account the occurrences of patch descriptors and assign a weight for patch descriptors based on their distance to the cluster centroid of the database in the feature space. First, it adopts a *K*-means method to cluster database descriptor sets ${\left\{f_{i}^{db}\in R^{1\times D}\right\}}_{i=1}^{N_{p}^{db}}$ to obtain the *K* centroid ${\left\{f_{i}^{c}\in R^{1\times D}\right\}}_{i=1}^{K}$. Then, the distance from a patch descriptor f to a centroid is calculated using the cosine distance to indicate its weighting factor $w_{c}\left(f\right)$ as [12]
  (2) $$w_{c}\left(f\right)=\sum_{i}^{\alpha }{\left\{d_{\cos}\left(f,f_{i}^{c}\right)\right\}}_{\min_{\alpha }},$$
  where ${\left\{\right\}}_{\min_{\alpha }}$ represents a subset of α smallest items.
- Secondly, a saliency context-driven weighting rule is proposed by assigning weights to patches within a feature map based on their variance. The underlying principle is that the variance within a patch indicates its significance or distinctiveness. Patches with higher variance might contain more salient information about the scene for more accurate place recognition. The weighting factor $w_{s}\left(f_{r,c}\right)$ of each patch feature is defined as
  (3) $$w_{s}\left(f_{r,c}\right)=\frac{1}{|\Omega_{r,c}|}\sum_{\left(m,n\right)\in \Omega_{r,c}}{\left(f_{m,n}-\bar{f}\right)}^{2},$$
  where $\bar{f}$ represents the mean feature vector averaged by all neighboring features $f_{m,n}$ centered at position (r,c), where $|\Omega_{r,c}|$ is the cardinality of the set of neighboring patches $\Omega_{r,c}$. By assigning higher weights to patch descriptors with high variance, two patch descriptors with greater significance contribute more to the place recognition task.

It is important to highlight the difference between the proposed context-driven patch matching and the conventional approaches [11,12]. Firstly, unlike the conventional approach [11], where all patches are treated as equally important and assigned the same weights during patch-wise matching, the proposed approach automatically evaluates the distinctiveness of patch features and assigns different weights accordingly. Secondly, while the approach [12] only considers a cluster-based weight (i.e., (2)) to exploit the global information from the whole database, the proposed approach applies both a cluster context-driven weighting rule and a saliency context-driven weighting rule. This allows for the utilization of both global and local information to evaluate the distinctiveness of patch features.

### 3.3. Summary of the Proposed Contextual Patch-Netvlad

**Figure 2:**
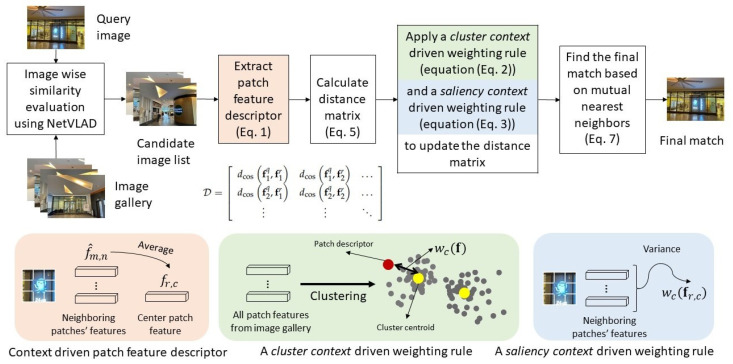
An overview of the proposed contextual patch-NetVLAD approach.

A summary of the proposed contextual patch-NetVLAD approach is provided in this section and illustrated in Figure 2.

First, we adopt NetVLAD [10] to find the top 100 images most similar to the query image and obtain the initial retrieval image set. The image pair list L is described as [11]

(4) $$L=\left(I_{q},I_{r}\right),$$

where $I_{q}$ and $I_{r}$ represent the query image and candidate image, respectively. For each image pair in L, the cosine distances (denoted as $d_{\cos}$) between two patch descriptors are calculated to generate the distance matrix D as [11]

(5) $$D=\left[\begin{array}{ccc} d_{\cos}\left(f_{1}^{q},f_{1}^{r}\right) & d_{\cos}\left(f_{1}^{q},f_{2}^{r}\right) & \ldots \\ d_{\cos}\left(f_{2}^{q},f_{1}^{r}\right) & d_{\cos}\left(f_{2}^{q},f_{2}^{r}\right) & \ldots \\ \vdots & \vdots & \ddots \end{array}\right].$$

Then, we update D by applying two proposed weighting mechanisms (2) and (3) on (5) to obtain

(6) $$D=\left[\begin{array}{ccc} \frac{1}{w_{c}\left(f_{1}^{q}\right)w_{s}\left(f_{1}^{q}\right)}\frac{1}{w_{c}\left(f_{1}^{r}\right)w_{s}\left(f_{1}^{r}\right)} & \frac{1}{w_{c}\left(f_{1}^{q}\right)w_{s}\left(f_{1}^{q}\right)}\frac{1}{w_{c}\left(f_{2}^{r}\right)w_{s}\left(f_{2}^{r}\right)} & \cdots \\ \frac{1}{w_{c}\left(f_{2}^{q}\right)w_{s}\left(f_{2}^{q}\right)}\frac{1}{w_{c}\left(f_{1}^{r}\right)w_{s}\left(f_{1}^{r}\right)} & \frac{1}{w_{c}\left(f_{2}^{q}\right)w_{s}\left(f_{2}^{q}\right)}\frac{1}{w_{c}\left(f_{2}^{r}\right)w_{s}\left(f_{2}^{r}\right)} & \cdots \\ \vdots & \vdots & \ddots \end{array}\right]\circ D,$$

where ∘ is the Hadamard product.

Next, we employ mutual nearest neighbors P, which represents a pair of the query image and database image that have nearest neighbors to each other in (6), to perform patch-level matching of the patch descriptors [11]

(7) $$P=\left\{\left(i,j\right):i=NN_{r}\left(f_{j}^{q}\right),j=NN_{q}\left(f_{i}^{r}\right)\right\},$$

where $NN_{r}\left(f\right)={\mathrm{argmin}}_{i}\left(d_{\cos}\left(f,f_{i}^{r}\right)\right)$ and $NN_{q}\left(f\right)=$${\mathrm{argmin}}_{j}\left(d_{\cos}\left(f,f_{j}^{q}\right)\right)$ retrieve the nearest neighbor descriptors matching the cosine distance in both query and reference image, respectively.

Finally, a spatial scoring method [11] is leveraged to compute the similarity score between a query/reference image pair, resulting in the final image retrieval results.

## 4. Experimental Results

### 4.1. Dataset

**Figure 3:**
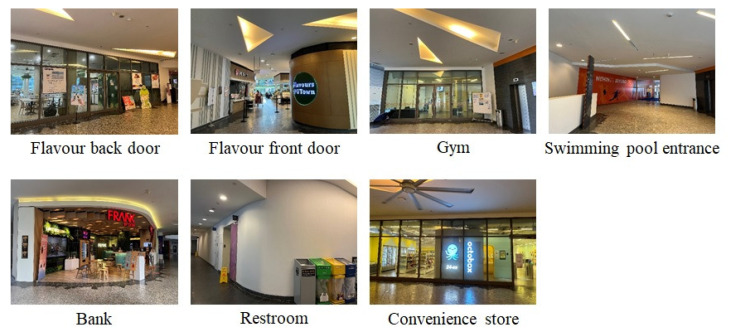
An overview of the benchmark dataset (UTown7) used in our experiments.

To conduct performance evaluation, we utilized two public benchmark datasets, Pittsburgh30k [13] and FMDataset [40], as well as a benchmark dataset UTown7 that we have collected ourselves (the UTown7 dataset is available at https://doi.org/10.17632/8td4f55j2g.1 (accessed on 1 November 2023). Our UTown7 dataset consists of images that are gathered from the Stephen Riady Centre, located on the campus of the National University of Singapore, as shown in Figure 3. This area was selected because it contains diverse indoor and outdoor environments that can create a rich image dataset for our study. We strategically chose seven unique locations across three different levels of the center, including *flavor back door, flavor front door, gym, swimming pool entrance, bank, restroom, convenience store,* to ensure the diversity of our collected images. All images are collected in varying light conditions and are of different architectural elements. For each location, we collected 20 raw images. Then, we applied data augmentation to generate an enlarged image dataset. Table 1 presents the list of data augmentation techniques applied, which include random cropping (extracting random patches from the original images), random flipping (randomly mirroring images horizontally), random rotation (applying arbitrary rotations to the images), random scaling (altering the size of objects in the images), random adjustments to brightness, contrast, and saturation (varying lighting conditions and color variations in the input data), and random noise (adding a degree of noise to images). The rationale behind using these parameters in data augmentation is to more accurately replicate the conditions under which people take photos in real-world situations. Leveraging these augmentation methods, we built a benchmark dataset with 1540 images to ensure that our model can be evaluated in different conditions, which is critical in the VPR performance evaluation.

### 4.2. Experimental Setup

In our experiments, all images were resized to a resolution of 640×480 pixels. Then, we extracted the patch-based features using a pre-trained patch-NetVLAD model [11] without any additional fine-tuning. The patch is defined as a size of 5×5 with a stride of 1. For the multiscale fusion, square patch sizes of 2, 5, and 8 were employed with associated weights of 0.45, 0.15, and 0.4, respectively. To provide a fair performance comparison, the same configuration was used for the model inference across our entire dataset. All approaches were implemented on a workstation equipped with an NVIDIA RTX A4000-16GB GPU, Intel(R) Xeon(R) CPU E5-2686 v4@2.30GHz, with Python 3.9, PyTorch 1.12.1, and CUDA 11.3.

Various VPR approaches were evaluated using the Recall@N metric, where a query image is graded to be correctly localized if at least one of the top *N* images falls within the ground truth tolerance range [10,41]. The recall rate is calculated as the percentage of query images that are correctly localized. We followed the strategy of patch-NetVLAD [11] to rank the initial retrieval top 100 image sets generated by NetVLAD [10]. In our experiments, we chose various *N* values to be 1, 5, and 10, respectively; therefore, the Recall@N metrics are Recall@1, Recall@5, and Recall@10.

### 4.3. Results

The proposed contextual patch-NetVLAD was evaluated with four state-of-the-art approaches, including NetVLAD [10], DELG [42], patch-NetVLAD [11], and patch-NetVLAD+ [12]. These methods were chosen due to their utilization of patch-level global descriptors as local features and their ability to re-rank patches. The experiments were conducted using two public benchmark datasets, Pittsburgh30k [13] and FMDataset [40], as well as a benchmark dataset that we have collected ourselves.

**Public benchmark datasets.** Our study evaluates five different methods on two public benchmark datasets, Pittsburgh30k [13] and FMDataset [40], as shown in Table 2. In each dataset, we randomly selected 500 images and further applied data augmentation to generate 5000 augmented images per dataset for the performance evaluation. Specifically, our method achieves Recall@10 of 99.82% on both Pittsburgh30k [13] and FMDataset [40], emphasizing the effectiveness of our data augmentation strategy in enhancing recognition accuracy. DELG [42] achieves moderate performance on both datasets, achieving Recall@1 of 49.11% and 54.65%, respectively. NetVLAD [10] and Patch-NetVLAD [11] demonstrated competitive performance, achieving Recall@5 as high as 97.45% on both datasets. Patch-NetVLAD+ [12] achieves Recall@1 (52.73%) on Pittsburgh30k [13], indicating enhanced precision for top-ranked retrievals.

**Our benchmark dataset.** The quantitative performance comparison of various VPR approaches using our benchmark dataset is shown in Table 3. Our proposed approach demonstrates superior performance compared to the other state-of-the-art methods, including DELG [42], NetVLAD [10], Patch-NetVLAD [11], and Patch-NetVLAD+ [12]. Specifically, our method achieves exceptional results on our proprietary dataset, showcasing a Recall@5 of 94.73% and a Recall@10 of 97.68%. These results exhibit substantial improvements of 6.46% and 6.01%, respectively, over Patch-NetVLAD [11]. Moreover, when compared to Patch-NetVLAD+ [12], our approach outperforms with an enhanced Recall@5 of 6.12% and a Recall@10 of 4.65%. Although our method’s Recall@1 is slightly lower than DELG [42] and NetVLAD [10], it surpasses all other methods in Recall@5 and Recall@10. The runtime taken to process one query image is 14.45 seconds because it applies a matching process to re-rank the top candidates in order to improve retrieval performance.

Our findings show noticeable differences between Recall@5 and Recall@10. The inferior Recall@1 performance might be due to the dataset characteristics. The augmentation process potentially introduces ambiguity into local features, affecting their distinctiveness and the clustering within their vicinity. Despite the decrease in Recall@1, both Recall@5 and Recall@10 show improvement. This is due to the increased diversity of the augmented dataset, which assists the model in capturing a wider range of features, consequently enhancing retrievals in distant neighborhoods.

### 4.4. Ablation Study

This section conducts ablation studies to assess the contributions of different components within our frameworks to the final recognition performance using our UTown7 dataset.

- To justify the proposed context-driven patch feature descriptor, we chose the patch-NetVLAD framework as the baseline approach. Then, we evaluated the performance of this framework by incorporating the proposed context-driven patch feature descriptor. Table 4 presents a quantitative performance evaluation using Recall@1, Recall@5 and Recall@10. The incorporation of the proposed context-driven patch feature descriptor leads to an accuracy improvement of 5.61% at Recall@5 and 5.27% at Recall@10, respectively.
- To justify the proposed context-driven patch-matching mechanism, we chose the patch-NetVLAD framework as the baseline approach. We then conducted a comparison of four variations, each incorporating two proposed rules: the proposed saliency context-driven weighting rule and the proposed cluster context-driven weighting rule. As shown in Table 5, the incorporation of both of the rules achieves the highest accuracy, with a recall rate of 94.73% at Recall@5 and 97.68% at Recall@10.

### 4.5. Discussion and Limitations

The proposed approach utilizes a sliding window technique to extract patch descriptors from NetVLAD, forming the foundation for a robust VPR system. Previous VPR methods such as Patch-NetVLAD and Patch-NetVLAD+ have employed NetVLAD for image retrieval but have struggled to balance local and global descriptor emphases, which impact accuracy and discriminative power. To counter these issues, the proposed approach enhances the approach with context-aware patch descriptors and matching mechanisms. It introduces two innovative modules: a context-aware patch descriptor and a context-aware patch-matching mechanism. The sliding window technique is used to extract patch descriptors, emphasizing the use of local regions for matching by breaking down the image into patches.

- The key strength of the proposed method lies in its ability to tackle the inherent challenges of VPR. The introduction of a context-aware patch descriptor ensures that the system captures both local and global information, mitigating the limitations of previous methods. Furthermore, the context-aware patch-matching mechanism enhances the discriminative power of descriptors extracted from locally similar regions in the descriptor space.
- The proposed approach potentially yields the following limitations. The first one lies in the challenge of balancing local and global descriptor emphases. Our method uses both local and global information, which could be challenging to balance effectively. This could impact the accuracy and discriminative power of the system. The second one lies in the computational complexity because the proposed approach applies a refinement pass (matching) to re-rank the top candidates in order to improve retrieval performance.

## 5. Conclusions

A new visual place recognition approach has been proposed in this paper by integrating two proposed modules into the conventional patch-NetVLAD framework to form a contextual patch-NetVLAD approach. Our approach achieves more accurate place recognition results due to the incorporation of two key components: the proposed context-aware patch descriptor, which aggregates features from neighboring patches, and the proposed patch-matching mechanism, which assigns different weights to guide the contribution of various patches. The proposed approach is able to achieve more accurate place recognition results, as verified in our quantitative performance comparisons using two public benchmark datasets and our benchmark dataset. In the future, we intend to apply our contextual patch-NetVLAD on a larger indoor dataset or a mixed indoor–outdoor dataset to further evaluate its performance and usage.

## Figures and Tables

**Table 1:** The list of image augmentation methods that are used to create the benchmark dataset (UTown7) for the performance evaluation of various VPR approaches.

Method	Detailed Parameter Settings
Random crop	[0.6,1]
Random flip	True or False
Random rotate	[−50,50]
Random scale	[0.8,1.2]
Random brightness, contrast, saturation	[−0.3,0.3]
Random noise	[0,255]

**Table 2:** The quantitative performance comparison of various VPR approaches on public benchmark datasets.

Method	Pittsburgh30k [13]			FMDataset [40]		
	**Recall@1**	**Recall@5**	**Recall@10**	**Recall@1**	**Recall@5**	**Recall@10**
DELG [42]	49.11	95.28	97.19	54.65	95.77	98.23
NetVLAD [10]	50.82	97.45	99.73	55.36	96.64	99.64
Patch-NetVLAD [11]	50.82	97.45	99.64	55.36	97.27	99.82
Patch-NetVLAD+ [12]	52.73	96.73	99.63	55.64	96.64	99.73
Ours	50.82	97.09	99.82	55.36	97.52	99.82

**Table 3:** The quantitative performance comparison of various VPR approaches on our UTown7 dataset.

Method	Recall@1	Recall@5	Recall@10
DELG [42]	78.52	87.46	90.19
NetVLAD [10]	79.76	89.12	92.52
Patch-NetVLAD [11]	79.76	88.27	91.67
Patch-NetVLAD+ [12]	79.76	88.61	93.03
Ours	76.53	94.73	97.68

**Table 4:** The ablation study of the proposed context-driven patch feature descriptor.

	Recall@1	Recall@5	Recall@10
Baseline	79.76	88.27	91.67
+ Proposed context-driven patch feature descriptor	79.76	93.88	96.94

**Table 5:** The ablation study of the proposed context-driven patch matching mechanism.

Proposed *Cluster* Context-Driven Weighting Rule (2)	Proposed *Saliency* Context Driven Weighting Rule (3)	Recall@1	Recall@5	Recall@10
-	-	79.76	93.88	96.94
*√*	-	79.76	88.61	93.03
-	*√*	72.79	93.20	97.32
*√*	*√*	76.53	94.73	97.68

## Data Availability

Data are contained within the article.
